# Comparison of the Removal of Synthetic Wastewater Samples Containing Basic Blue 3 Dye Using Electrochemical and Adsorption Methods

**DOI:** 10.3390/molecules30204039

**Published:** 2025-10-10

**Authors:** Beyza Moralı, Türkan Börklü Budak

**Affiliations:** Department of Chemistry, Faculty of Art and Science, Yıldız Technical University, 34220 İstanbul, Türkiye; beyza.morali@std.yildiz.edu.tr

**Keywords:** basic blue 3, adsorption, linden leaves, *Tilia* L., electrocoagulation (EC)

## Abstract

Water pollution, a significant environmental issue, is growing more urgent. This study evaluated the effectiveness of adsorption and electrocoagulation methods in removing Ba-sic Blue 3 (BB3), a common dye used in the textile industry, from water. For the adsorption process, linden tree leaves—often used for health benefits in existing literature—were employed, while in the electrocoagulation (EC) method, an aluminum electrode was used. The results show that the optimal conditions for adsorption were an initial BB3 concentration of 5 mg/L, 50 mL of 0.9 g *Tilia* L. adsorbent, 60 min, 180 rpm, 30 °C, and pH 10, achieving a removal efficiency of 99.21%. The optimal conditions for electrocoagulation were 1 L of 15 mg/L initial BB3, a current density of 2.64 mA/cm^2^, 15 mL of 0.2 M KCl, a reaction time of 90 min, a stirring speed of 100 rpm, and a pH of 10, resulting in a removal efficiency of 97.98%. The results indicate that linden leaves, a natural and sustainable material, showed a slightly higher removal percentage (99.21%) in the EC method over a shorter period (60 min). Conversely, the EC method also achieved a significant removal rate (97.98%, 90 min). In summary, both methods demonstrate strong BB3 removal capabilities and could help improve wastewater treatment processes.

## 1. Introduction

Synthetic dyes are used in textile maintenance, as well as in cosmetics, printing, pharmaceuticals, and other industries like food processing [1]. Synthetic dyes, which are often not properly treated before being released by various industries, create serious environmental issues by polluting water bodies such as lakes, rivers, and oceans [2]. Cationic dyes are particularly harmful because they are highly toxic, carcinogenic, and non-biodegradable [3]. Among these, the BB3 cationic dye, frequently found in the environment, causes oxygen deficiency by preventing the generation of solar radiation needed for photosynthesis by aquatic organisms. It can also cause serious waterlogging and affect other organisms [4,5].

Because dyes are not biodegradable, wastewater containing dyes requires physical or chemical treatment methods such as adsorption, coagulation, sedimentation, filtration, photocatalysis, electrochemical treatment, and chemical oxidation [6,7].

Among these, the electrochemical treatment method offers several advantages over traditional methods [8]. A review of the literature demonstrates the effective role of the EC method in removing ketoprofen (KTP) from its aqueous counterpart, combining electrocoagulation (EC) and adsorption methods [9]. Another effective method, this time using zeolite and ultrasound-assisted EC, successfully removed toxic substances from wastewater [10]. Similarly, in a study on plant pigments, it was reported that electrocoagulation exhibited varying efficiencies depending on the solvent medium, but phenolic compounds and tannins could be effectively removed in aqueous systems [11]. Furthermore, it was noted that increasing the NaCl electrolyte concentration not only resulted in high success in dye removal (up to 93% for tartrazine) but also provided efficiency in hydrogen production [12]. These findings demonstrate that electrocoagulation, when applied alone or in combination with methods such as adsorption, is a promising technology for both reducing environmental pollution and producing sustainable energy.

Another frequently used treatment method is the adsorption technique. It has promising potential for the removal of dyes from wastewater due to its ease of application, low cost, and high performance [13,14,15,16]. Activated carbon, one of the available adsorbents, is one of the most effective adsorbents used in wastewater treatment due to its high adsorption capacity for organic matter [17,18]. In another study, sawdust obtained from linden trees was used as an adsorbent to remove heavy metal ions from the environment [19]. Linden tree leaves, included in this study, have many benefits in the medical literature. They are widely utilized for their flavonoid content, quercetin, and kaempferol, which are associated with anti-inflammatory and anti-swelling properties [20]. The presented study investigated the effectiveness of adsorption and electrochemical treatment methods in removing the BB3 cationic dye from aqueous media.

## 2. Result and Discussion

### 2.1. Characterization of Linden Leaf Adsorbent

#### 2.1.1. SEM Analysis of the Adsorbent Material

**Figure 1:**
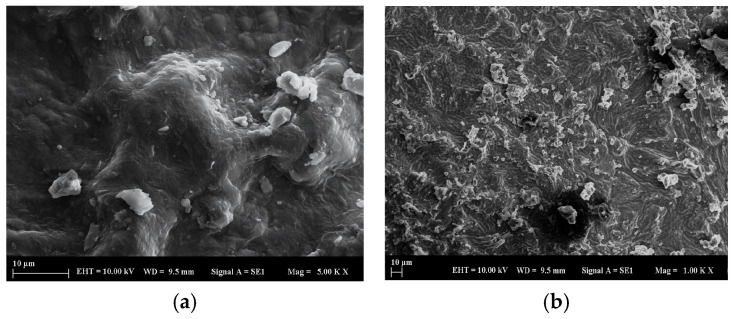

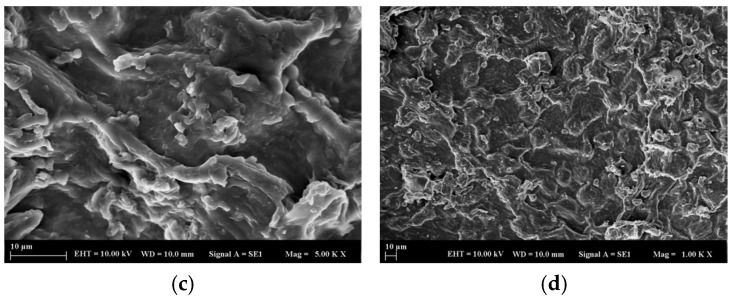
SEM images of linden leaves before (**a**,**b**) and after (**c**,**d**) adsorption.

To clarify the morphological properties of the adsorbent made from linden leaves before and after treatment with BB3 dye, measurements were taken using a scanning electron microscope (SEM). As shown in Figure 1a,b, before dye adsorption (Figure 1a: 5.0 KX magnification, EHT 10.0 kV, WD 9.5 mm, and particle size 10 µm; Figure 1b: 1.0 KX magnification, EHT 10.0 kV, WD 9.5 mm, and particle size 10 µm), it is evident that the accumulation surface increases after adsorption (Figure 1c,d) due to the areas available for accumulation on the surface before adsorption. In this figure, the rise in surface accumulation supports the increase in % removal values.

#### 2.1.2. Analysis of FTIR Results

FT-IR spectra enable the comparison of samples before and after adsorption. Generally, the band positions remain essentially unchanged, but noticeable differences in intensity and width are apparent. Notably, the broader and more intense bands in the 3200–3600 cm^−1^ region in the “after” spectrum indicate that surface hydroxyl groups have become more prominent, likely due to hydrogen bonding interactions with dye molecules. Similarly, aliphatic C–H stretching vibrations are observed in both spectra within the 2920–2850 cm^−1^ range, with increased intensity in the post-adsorption spectrum, supporting the interaction of dye-related aliphatic groups with the surface. The carbonyl (C=O) band, initially weak around 1700 cm^−1^ in the “before” spectrum, becomes significantly stronger after adsorption, suggesting that carbonyl groups in the dye are binding to the adsorbent surface. The sharper, more intense aromatic C=C vibrations in the 1600–1500 cm^−1^ range after adsorption suggest that the dye’s aromatic structure is attached to the surface. Additionally, the increased intensity of bands linked to C–N and C–O stretching vibrations in the 1000–1300 cm^−1^ region confirms that specific dye functional groups interact with the adsorbent surface. These spectral changes indicate that the observed interactions are surface interactions rather than permanent structural modifications to the adsorbent, confirming that the dye has successfully adhered to the surface.

**Figure 2:**
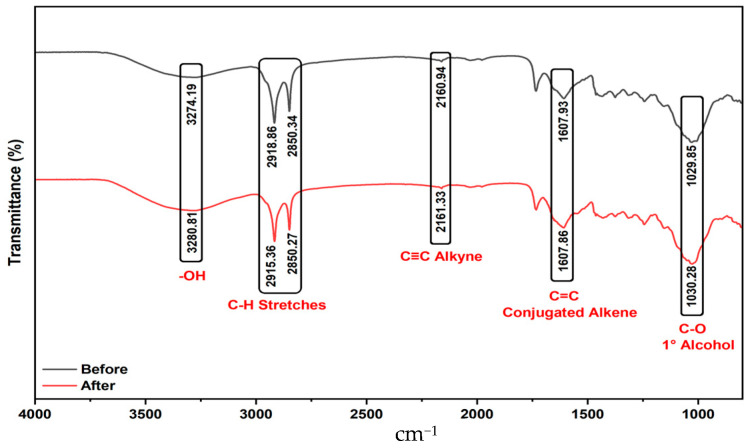
The FTIR-ATR spectra of *Tilia* leaves powder before and after adsorption of BB3.

As shown in Figure 2, comparing the FTIR-ATR measurement results before and after adsorption reveals that the molecular vibrations in the functional groups remain consistent. Therefore, in line with the SEM morphological characterization findings, it is understood that adsorption occurs through surface adhesion and does not alter the chemical composition of the adsorbent. This suggests that adsorption mainly occurs through physical mechanisms, such as van der Waals forces, electrostatic attraction, or hydrogen bonding.

#### 2.1.3. XRD Results

**Figure 3:**
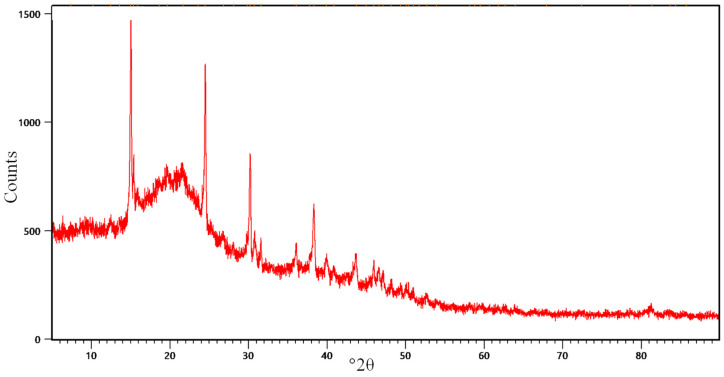
XRD data of linden leaf adsorbent.

To clarify the cellular components of linden leaf powder, the XRD spectra shown in Figure 3 were analyzed. Accordingly, the values determined at °2θ in the spectrum are 15.02°, 21.64°, 24.49°, 30.22°, 38.33°, 43.73°, 45.97°, and 50.38°, respectively. The greater number of sharp peaks in the spectrum indicates a more prominent crystalline nature.

#### 2.1.4. BET Analysis of Linden Leaf Adsorbent

**Figure 4:**
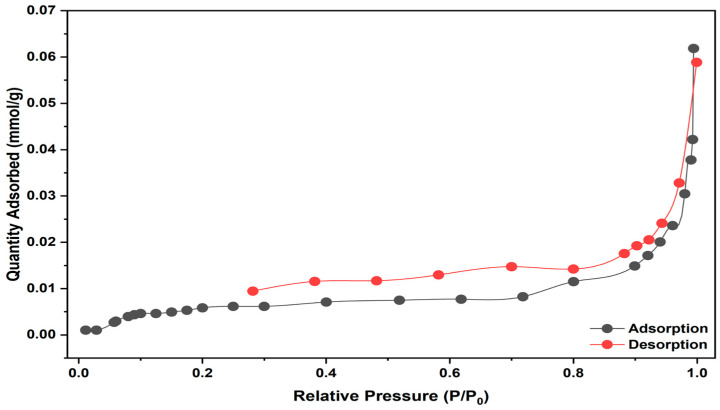
The BET pattern of linden leaves.

The results of BET measurements used to determine the textural properties of the linden leaf adsorbent are presented in Figure 4. The surface area was found to be 0.08 m^2^/g, the pore volume was 0.0003 cm^3^/g, and the pore width was 14.66 nm. According to the IUPAC pore size classification, pores are categorized as ultramicroporous (<0.7 nm), microporous (0.7–2 nm), mesoporous (2–50 nm), and macroporous (>50 nm) [21].

It is understood that the linden leaf adsorbent has a “mesoporous” structure with a measurement of 14.66 nm. In addition, nitrogen adsorption–desorption isotherms confirming the Type IV hysteresis loop characteristic [22] are notable in Figure 4.

#### 2.1.5. pH_pzc_ (Point of Zero Charge) Determination

**Figure 5:**
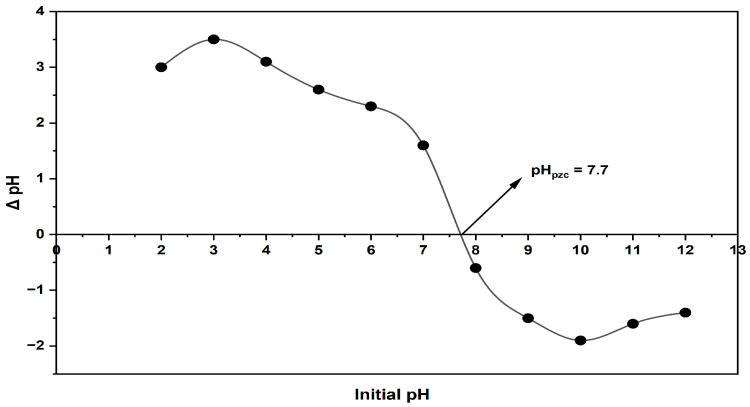
pH_pzc_ graph of linden leaf adsorbent.

The pH_pzc_ value of the linden leaves used is shown in Figure 5. At pH values below this threshold, the repulsive force of the cationic dye molecules increases due to the rising H^+^ concentration, resulting in a decrease in the percentage of removal. The results from experiments conducted at different pH levels in the following sections support this, with the highest % removal value occurring at pH 10 when pH > pH_pzc_.

### 2.2. The Results of Adsorption Experiments

The results of the studies to determine the optimal working conditions for the adsorption experiments conducted using linden leaves prepared as an adsorbent are given below.

#### 2.2.1. The Effect of Initial Dye Concentration on Adsorption of BB3

**Figure 6:**
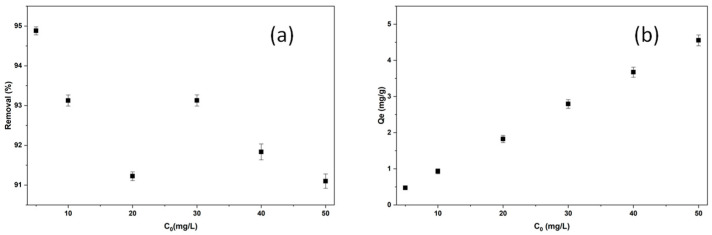
The removal efficiency (**a**) and adsorption capacity (**b**) results of BB3 at different initial dye concentrations.

The initial dye concentration, a parameter influencing removal efficiency, was examined at concentrations from 5 to 50 mg/L. For BB3, the amount of adsorbent was fixed at 0.5 g, while all other parameters remained constant: V = 50 mL, pH approximately 6.5, 150 rpm, 25 °C, and 60 min. The highest removal efficiency of 94.88% was achieved at the initial concentration of 5 mg/L. % removal and Q_e_ values are shown in Figure 6a,b.

The percentage removal values in the presented study ranged from 94.88% to 91.22%, while Q_e_ values varied between 0.5 and 4.5 mg/g. Analysis of the data revealed that the highest removal capacity for BB3 molecules in the medium was 94.88% at a concentration of 5 mg/L, corresponding to the saturation point. It is believed that the different percentage removal values at higher concentrations may result from uneven saturation of dye molecules on the adsorbent surface, leading to variations in removal efficiency.

#### 2.2.2. The Effect of Adsorbent Dosage on Adsorption of BB3

One of the main factors affecting adsorption and removal efficiency is the amount of adsorbent used. Percent removal experiments for BB3 dyes were conducted using *Tilia* leaves, which were ground into a powder. The C_0_ value for BB3 was set at 5 mg/L. Other parameters remained constant at V = 50 mL, pH around 6.5, 150 rpm, 25.0 °C, and 60 min. The amount of adsorbent ranged from 0.1 g to 1.5 g, and the experiments were repeated.

**Figure 7:**
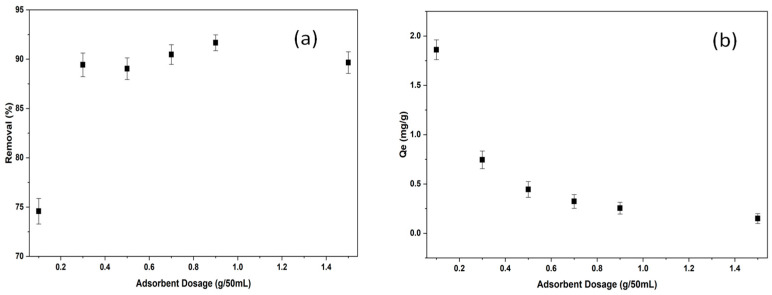
The removal efficiency (**a**) and adsorption capacities (**b**) results of BB3 at different adsorbent dosages.

As shown in Figure 7a, the percentage removal values ranged from 74.34% to 91.67%. Analyzing Figure 7b reveals that Q_e_ values varied between 0.13 and 1.87 mg/g. The results indicate that the highest removal efficiency for BB3 dye was 91.67% with 0.9 g of adsorbent.

When the amount of adsorbent increases to a certain point, the active surface area and binding sites required for dye removal increase, thereby improving the adsorption process and enhancing the percentage removal rate. However, once the adsorbent amount exceeds this point, particles tend to clump together due to the excess material, which decreases the active surface area. Additionally, when more adsorbent is used relative to a constant contaminant level in the solution, the surface area of the adsorbent might not be fully utilized. For these reasons, percentage removal rates are likely to decrease when the adsorbent amount goes beyond the optimal level.

#### 2.2.3. The Effect of Contact Time and Agitation Rate on Adsorption of BB3

The effect of contact time on the adsorption process was evaluated at various contact times, ranging from 5 to 180 min, while other factors, such as pH ≅ (6.5) and 150 rpm, remained constant. The results, obtained by increasing the initial concentration to 5 mg/L with 0.9 g/50 mL of adsorbent for the BB3 dye, ranged from 89.55% to 96.06%.

**Figure 8:**
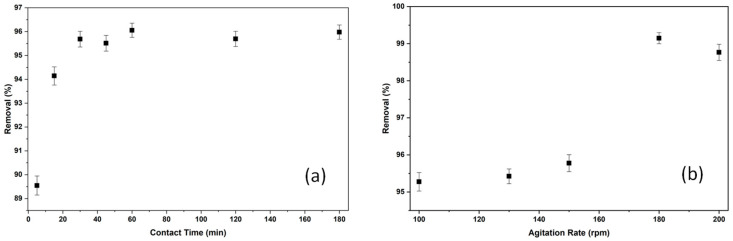
The removal efficiencies of contact time (**a**) and agitation rate (**b**).

The effect of shaking speed on adsorption was investigated at speeds ranging from 100 to 200 rpm. For the BB3 dye, the conditions of 0.9 g of *Tilia* tree leaves, an initial concentration of 5 mg/L, a 60 min contact time, an ambient temperature of 25 °C, and a pH ≅ of approximately 6.5 were kept constant. The highest adsorption of 99.15% occurred at 180 rpm, as shown in Figure 8b.

As shown in Figure 8a, the removal of BB3 dye by the adsorbent started at minute 15 and gradually increased over time. It reached its peak at minute 60. This indicates that the adsorbent held onto the dye molecules and an equilibrium was reached. Adsorption efficiency slightly declined after minute 60, likely due to desorption during the extended period, which resulted in a small drop in % removal values. Nonetheless, the optimal removal occurred at minute 60 with a removal rate of 96.06%.

The adsorption of BB3 dye on the adsorbent material increased from 95.3% to 99.15%. This rise in percentage removal, caused by increasing agitation speed, is believed to improve the adsorption rate by making the film layer around the adsorbent particles more receptive to adsorption. The optimal removal rate at 180 rpm decreased slightly to 98.68% at the higher agitation speed of 200 rpm. This is likely due to increased physical repulsion, which causes desorption and a slight decrease in percentage removal.

#### 2.2.4. The Effect of Temperature and pH on the Adsorption of BB3

**Figure 9:**
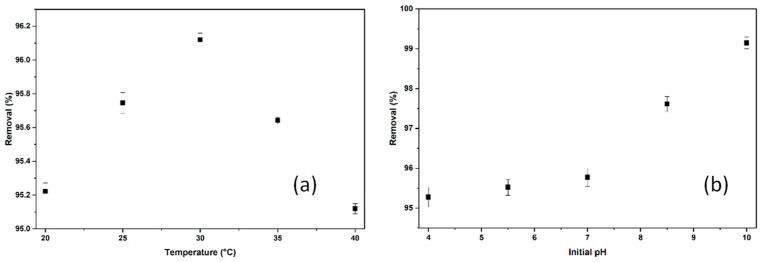
The removal efficiencies of temperature (**a**) and initial pH (**b**).

The effect of ambient temperature on the adsorption process was assessed at various temperatures between 20 °C and 40 °C, while other parameters, such as pH ≅ 6.5 and 180 rpm, remained constant. For BB3 dye, results ranged from 95.12% to 96.12% using the parameters of 0.9 g of adsorbent, an initial concentration of 5 mg/L, and a contact time of 60 min. The optimal temperature was 96.12%, as shown in Figure 9a.

As shown in Figure 9a, despite the increase in temperature during the experiments, there is no significant change in the percentage of removal. It is believed that the adsorption process occurs through low-energy barriers involving physical interactions and thus does not cause notable changes in % removal across the tested temperature range. This suggests that the process may operate via a stable mechanism that is not highly influenced by temperature.

Buffer solutions with pH levels from 4 to 10 were used to determine the initial pH. For BB3 dye, the parameters were set as follows: 0.9 g of *Tilia* tree leaves, an initial concentration of 5 mg/L, a 60 min contact time, a 180 rpm shaking speed, and an ambient temperature of 30 °C. According to the results in Figure 9b, an optimal initial pH of 10 achieved a 99.23% success rate.

When the experimental data were analyzed, pH_pzc_ = 7.7 was identified. As shown in Figure 9b, the removal percentage at pH values below the critical point ranged from 95.2% to 97.5%. It is believed that the increased H^+^ charge on the adsorbent surface at these pH levels could activate the cationic dye BB3 in a repulsive manner. At pH 10, which is greater than the pH_PZC_ of 7.7, a removal efficiency of 99.21% was achieved, indicating that the optimal pH was found to be 10.

#### 2.2.5. Regeneration of the Adsorbent

**Figure 10:**
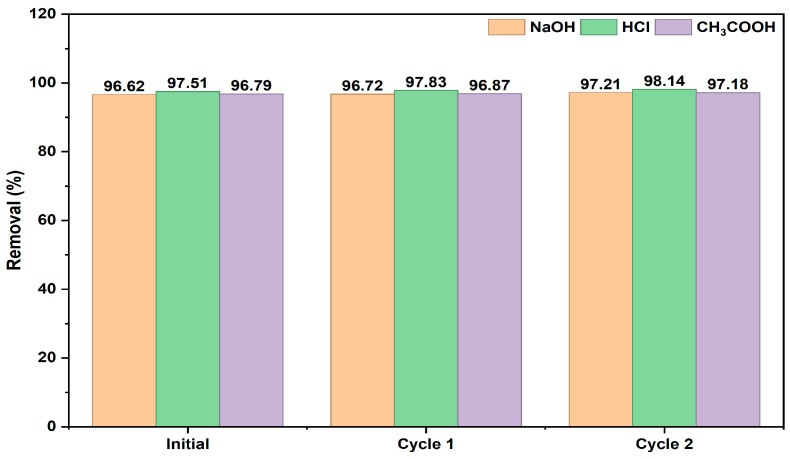
The regeneration performance of the adsorbent over two cycles of adsorption and desorption using BB3 dyes: 0.1 M NaOH, 0.1 M HCl, and 0.1 M CH_3_COOH.

To apply the regeneration process to the adsorbent obtained from linden leaves, 0.1 M HCl, NaOH, and CH_3_COOH solutions were used for elution. After achieving optimal adsorption, the adsorbent was treated separately with the indicated solutions to regenerate it. Before reuse, the adsorbent was rinsed three times with ultrapure water and dried in an oven at 65 °C for 24 h to achieve a stable mass. Thus, the regenerated linden leaf adsorbent was ready for subsequent BB3 adsorption tests. The results from two replicates are shown in Figure 10.

According to Figure 10, it is evident that after two repeated regenerations, each eluent solution yields results that support the reusability of the adsorbent.

#### 2.2.6. Adsorption Isotherms

**Figure 11:**
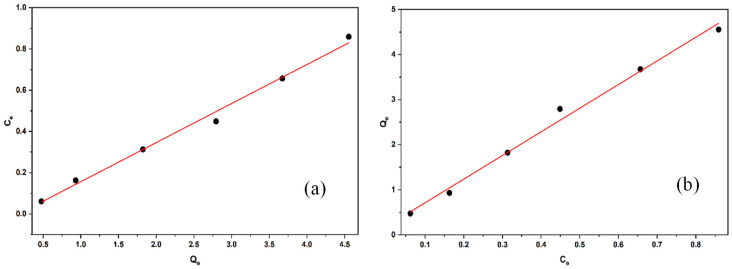
The isotherm plots of the linear Langmuir (**a**) and Freundlich (**b**) isotherms for the adsorption of BB3 dyes using linden leaves as an adsorbent.

The linear, Freundlich, and Langmuir adsorption isotherm models are plotted in Figure 11a and Figure 11b, respectively. The Langmuir and Freundlich isotherm parameters and corresponding r^2^ values calculated using Equations (3)–(5) are presented in Table 1. r^2^ values were used to identify the best-fitting isothermal adsorption model for linden leaves with BB3 dye. The model with the r^2^ value closest to 1 is chosen as the best fit.

Based on the data in Table 1, the r^2^ linear regression coefficients were 0.992 for the Langmuir isotherm and 0.993 for the Freundlich isotherm. The maximum adsorption capacity for BB3 was calculated as 5.283 mg/g. The results suggest that the linden leaf adsorbent has a uniform distribution in removing the BB3 dye and achieves a monolayer surface coverage.

#### 2.2.7. Comparative Research of Adsorption Capacity for BB3

Some studies on removing BB3 dye are listed in Table 2. In addition to being renewable, natural, and abundant, linden tree leaves have significant potential for BB3 removal. Based on this data, it is predicted that effective results can be achieved in removing BB3 dye from wastewater using an adsorbent made from linden tree leaves.

### 2.3. The Results of Electrocoagulation (EC) Experiments

The results of the studies aimed at identifying the optimal parameters for removing BB3 dye using the electrochemical method are presented below.

#### 2.3.1. The Effect of Different Concentrations of BB3 on % Removal

The removal of BB3 dye by electrocoagulation was studied at initial concentrations ranging from 2 to 20 mg/L (ppm). During these tests, the distance between the aluminum (10 × 5 cm) and graphite electrodes was 3 cm, the current density was 4.2 mA/cm^2^, the volume was 1000 mL, with 10 mL of 0.2 M KCl, and the stirring rate was 100 rpm at 25 °C.

**Figure 12:**
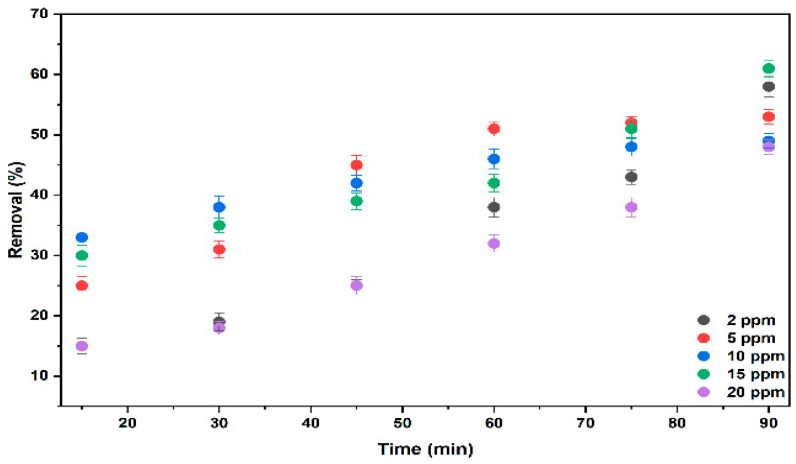
The removal efficiency graph of BB3 at various initial dye concentrations.

The effect of the initial BB3 concentration on the electrocoagulation process increases with higher dye levels, as shown in Figure 12. During electrolysis, Al^3+^ ions entering the solution react with OH^-^ ions, precipitating and forming Al(OH)_3_, a coagulant that helps in removing BB3. The highest removal efficiency observed was 61.11% in a solution with 15 mg/L BB3.

#### 2.3.2. The Effect of Different KCl and NaCl Volumes on the % Removal of BB3

**Figure 13:**
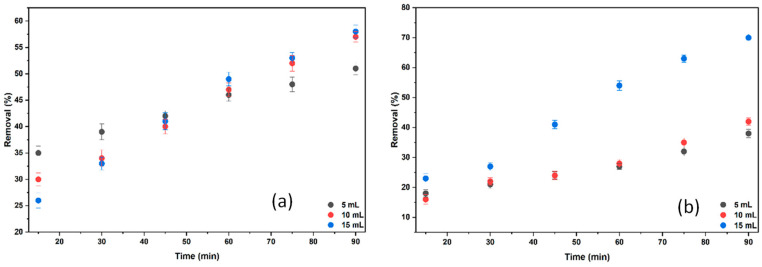
Examining the effects of different electrolytes on removal efficiency (**a**) 0.2 M NaCl; (**b**) 0.2 M KCl.

To examine the effect of electrolyte on the % removal values, electrolysis was performed by adding 5, 10, and 15 mL of 0.2 M NaCl solution. The results of the experiments using 1 L of BB3 solution at a concentration of 15 mg/L are shown in Figure 13a. The parameters used during these experiments included a 3 cm distance between electrodes, a volume of 1000 mL, a rotation speed of 100 rpm, and a temperature of 25 °C. The same procedures were followed with the addition of 15 mL of 0.2 M KCl solution. The results are presented in Figure 13b.

The type of electrolyte added to the solution is a key factor influencing the results of electrocoagulation. In Figure 13a, BB3 removal experiments with 15 mL of 0.2 M NaCl achieved a 58.04% removal, while those with 15 mL of 0.2 M KCl resulted in a 70.03% removal. The higher BB3 removal rate in experiments using KCl compared to NaCl is likely due to the lower hydration energy of K^+^ ions versus Na^+^, which leads to more stable Al(OH)_3_ flocs that enhance removal efficiency, causing a significant increase in BB3 removal.

#### 2.3.3. The Effect of Different Stirring Speed and pH on the % Removal of BB3

BB3 dye removal via electrocoagulation was repeated at rotation speeds from 100 to 300 rpm. Other operating parameters included 1 L, an initial concentration of 15 mg/L, 15 mL of 0.2 M KCl, and an ambient temperature of 25 °C.

**Figure 14:**
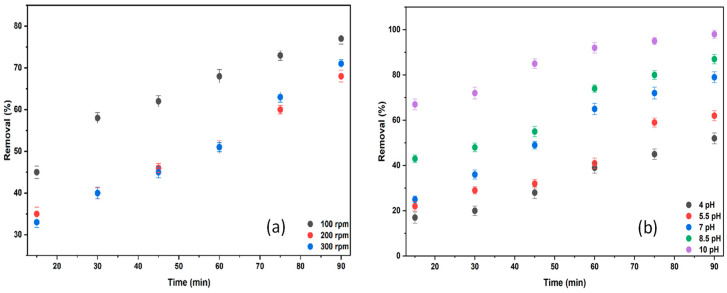
The effect of stirring rate (**a**) and pH (**b**).

Furthermore, tests were repeated across a pH range of 4–10 to determine the optimal pH value. Other operating conditions included 1 L, an initial concentration of 15 mg/L, 15 mL of 0.2 M KCl, an ambient temperature of 25 °C, and a stirring rate of 100 rpm. The results are presented in Figure 14b.

**Figure 15:**
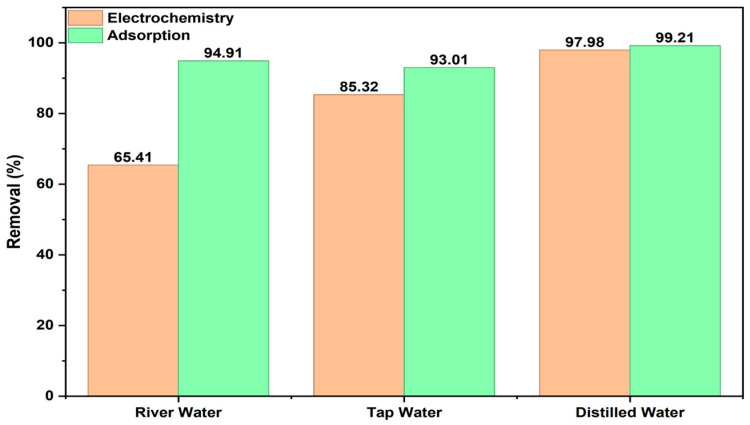
The results of BB3 solutions prepared from different water sources.

In electrocoagulation experiments, once the optimal rotation speed was reached, the flocs responsible for dye removal due to Al^3+^ ions were evenly distributed, and the removal rate was maximized. As shown in Figure 15, the highest BB3 removal was 77.02% at 100 rpm. Conversely, lower removal percentages resulted from the mechanical degradation of the flocs formed at higher rotation speeds.

As shown in Figure 14b, the removal of BB3 reaching 97.98% at pH 10 was identified as the optimal value. During electrocoagulation, the concentration of Al(OH)_4_^−^ ions in the solution increases at higher pH levels due to the migration of ions from the Al electrode. This is believed to positively influence the removal of the cationic dye BB3.

#### 2.3.4. Different Electrode Studies in the Literature

When reviewing literature data, the studies conducted with different electrode types are listed in Table 3. As in other studies involving the Al electrode used in the electrocoagulation method, it is observed that successful results can be achieved, with a removal rate of 97.98% for BB3.

### 2.4. Experimental Studies by Using BB3 Solutions Prepared from Different Water Sources

The experiments conducted so far used solutions made with distilled water, following laboratory conditions. However, to assess how different water sources affect the results, BB3 dye solutions were also prepared with tap water and river water. The percentage removal performance was then tested using adsorption and electrocoagulation methods, based on the determined optimum operating parameters.

The optimal operating parameters for the adsorption method are as follows: 50 mL of a solution containing 5 mg/L BB3, 0.9 g of adsorbent, 60 min of shaking at 180 rpm, at 30 °C, and a pH of 10. The optimal conditions for electrocoagulation are: 1 L of solution containing 15 mg/L BB3, 15 mL of 0.2 M KCl, 90 min of stirring at 100 rpm, at 25 °C, and a pH of 10. The results are shown in Figure 15.

As shown in Figure 15, studies involving BB3 using the electrochemical method revealed removal rates of 65.41%, 85.32%, and 97.98% with river water, tap water, and distilled water, respectively. Similarly, when the adsorption method was applied, removal rates of 94.91%, 93.01%, and 99.21% were achieved with the same water types. The decrease in removal efficiency observed with river water and tap water in the electrocoagulation studies may be attributed to the presence of different anion and cation impurities in the water sources. Nonetheless, a substantial removal percentage was still achieved. The removal rates obtained with the adsorption method demonstrate that removal rates above 90% were successfully accomplished across all three water sources.

## 3. Experimental Section

### 3.1. Materials

Basic Blue 3, a cationic dye, was obtained from Sigma-Aldrich (Merck KGaA, St. Louis, MO, USA) and used as purchased. (BB3; C_20_H_26_ClN_3_O, molecular weight = 359.89 g/mol, λ_max_ = 654 nm). The stock solution of BB3 dye was prepared with distilled water at a concentration of 1.0 g/L, and solutions were obtained by diluting at the desired ratios. pH 4, 7, and 10 buffer solutions, supplied by Acros Organics (Thermo Fisher Scientific, Geel, Belgium), are used to provide different pH values. Solutions with pH values of 4, 7, and 10 were used as citrate, phosphate, and carbonate buffer solutions, respectively. Chloride KCl and NaCl used for electrocoagulation experiments were supplied by Sigma-Aldrich (Merck KGaA, St. Louis, MO, USA). Graphite and aluminum electrodes were used as cathodes and anodes.

### 3.2. Instrumentation

A Julabo SW 22 (ISOLAB Laborgeräte GmbH, Eschau, Germany) heated shaking water bath was used for batch adsorption experiments. During these experiments, samples were taken from the solution and analyzed against a blank solution using a UV-VIS spectrophotometer (Agilent 8453, 190–1100 nm, Agilent Technologies, Santa Clara, CA, USA) with a 1 cm quartz cuvette. A Nicolet IS10 FTIR spectrometer (FTIR-ATR; Nicolet IS10, Thermo Fisher Scientific Inc., Waltham, MA, USA) was used to analyze the functional groups in the adsorbent structure. During measurement, the range was 4000–400 cm^−1^, with a resolution of 4 cm^−1^, and 32 scans were taken. The background was measured before each scan, and the ATR diamond crystal was used.

The distilled water used in the experiments was obtained from a Mikrotest MSD-08 purification system, 8 L, with a conductivity target of approximately 2.3 µS/cm (Mikrotest MSD-08, İstanbul, Türkiye). An Elektromag M615M centrifuge (Elektromag M615M, Ankara, Türkiye) was used for centrifugation. A Memmert oven, capable of reaching temperatures from +20 °C to +300 °C (Memmert GmbH + Co. KG, Schwabach, Germany), served as a heater during the necessary steps. A direct current power supply was connected to the electrolysis cell to provide power in the electrocoagulation experiments. Additionally, a comprehensive characterization of the adsorbent material was conducted using X-ray diffraction (XRD; Malvern PANalytical X’Pert PRO, Malvern, UK), with the following settings: 40 mA, 45 kV, a goniometer radius of 240 mm, and a focus-divergent slit distance of 144.50 mm. Morphological evaluation and crystallographic analysis were performed using a scanning electron microscope (SEM; Zeiss EVO LS10, Oberkochen, Germany), with the following settings: 10 µm, EHT = 10.00 kV, WD = 9.5 mm, Signal A = SE1, Mag = 5.00 KX and 1.00 KX. The textural properties of the adsorbent (pore volume, specific surface area) were determined through nitrogen physical adsorption measurements using the Brunauer–Emmett–Teller (BET) method (Micromeritics ASAP 2020, Norcross, GA, USA), with the following parameters: N_2_ adsorption analysis, analysis bath temperature of −195.441 °C, and an equilibrium interval of 10 s.

### 3.3. Preparation of Linden (Tilia) Tree Leaves as the Adsorbent

**Figure 16:**
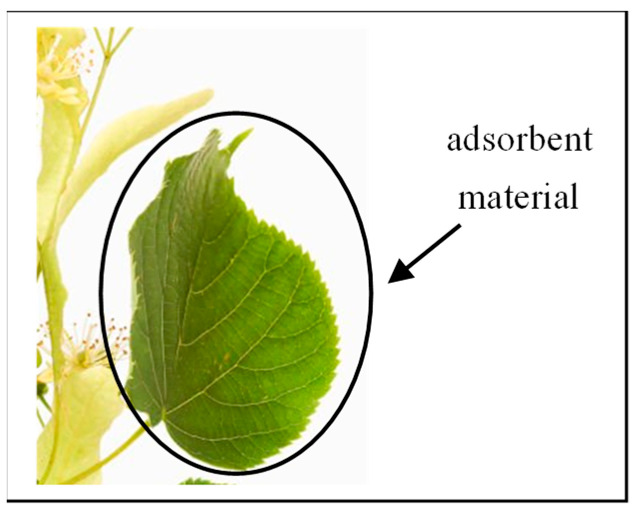
Linden (*Tilia*) tree leaves are used as an adsorbent material.

In this study, the large leaves of the Linden (*Tilia*) tree, shown in Figure 16, were used as adsorbents. The leaves were collected from İstanbul, Türkiye. To remove foreign particles, the adsorbent was washed with tap and distilled water. After drying in an oven at 80 °C, the material was ground into a fine powder using a mechanical blender and then pulverized to a 60–80 mesh size.

### 3.4. Adsorption Experiments

**Figure 17:**
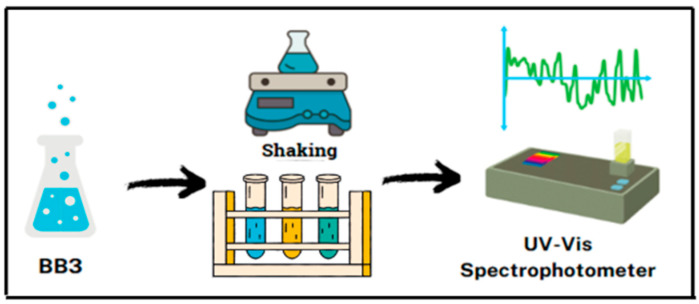
Adsorption process.

The batch adsorption method was used to assess the adsorption capacity of aqueous solutions of BB3 cationic dye using an adsorbent derived from *Tilia* tree leaves. Solutions of different concentrations were prepared by diluting stock BB3 dye solutions. Then, 50 mL of these standard solutions at initial concentrations were placed in 100 mL beakers and stirred in a water bath. During the experiments, the effects of parameters such as initial dye concentration (5–50 mg/L), adsorbent amount (0.1–1.5 g), contact time (5–180 min), temperature (25–40 °C), shaking speed (100–180 rpm), and pH (4–10) were examined. After adsorption, the samples were first centrifuged at 5000 rpm for 3 min, filtered through filter paper, and then transferred to test tubes. The absorbance was measured at λ_max_ (654 nm for BB3) using a UV-VIS spectrophotometer, and the results were recorded (Figure 17).

The following equations were used to calculate the percentage removal (%R) and adsorption capacity (*Q_e_*) of BB3 dyes in solution:

(1) $$Removal\;\left(\%\right)=\frac{C_{0}-C_{e}}{C_{0}}\times 100$$

(2) $$\;\;\;Q_{e}=\frac{C_{0}-C_{e}}{m}\times V$$

In the equations, $C_{0}$ is the initial dye concentration and $C_{e}$ is the dye concentration at time t (mg/L), m is the amount of adsorbent (g), and V is the volume of the dye solution (L).

### 3.5. The Figureout Procedure of pH_pzc_ (Point of Zero Charge)

The drift method, often used in research to determine the pH_pzc_ value of the linden leaf adsorbent because of its simplicity and reliability, was applied in this study [35]. For this experiment, 40 mL of 0.1 M NaNO_3_ solution was poured into eleven 100 mL glass beakers. The initial pH values (pH_i_) of the beakers were adjusted to range from 2 to 12 using 0.1 M HCl or 0.1 M NaOH solutions. Each beaker received 0.5 g of adsorbent and was stirred at room temperature for 48 h to reach equilibrium. At the end of this period, the final pH (pH_f_) of each solution was measured with a pH meter. When creating graphs, the initial pH (pH_i_) was plotted on the x-axis, and the change in pH (ΔpH = pH_f_ − pH_i_) was plotted on the y-axis.

### 3.6. The Isotherms of Adsorption

The optimization and design mechanisms of adsorption systems are based on adsorption isotherms [36]. The equations of the linear Langmuir and Freundlich isotherm models applied in the study are shown in Equations (3)–(5):

(3) $$\;\;\frac{C_{e}}{Q_{e}}=\frac{1}{K_{L}Q_{max}}+\frac{C_{e}}{Q_{max}}$$

(4) $$\;\;\;\;\;\;\;\;\;\;\;\;\frac{1}{Q_{e}}=\frac{1}{K_{L}Q_{max}}\times \frac{1}{C_{e}}+\frac{1}{Q_{max}}$$

(5) $$\;\;\;\;\;\;\;\;\;\;\;\;\;\;LogQ_{e}=LogK_{f}+\frac{1}{n}LogC_{e}$$

### 3.7. Electrocoagulation (EC) Experiments

**Figure 18:**
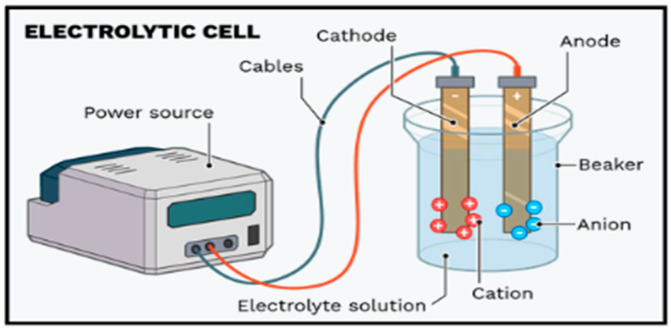
Electrocoagulation process.

The working solution was created by diluting the stock BB3 solution at suitable ratios. Solutions at the specified concentrations were placed in a 1000 mL electrolysis cell. Graphite (cathode) and aluminum (anode) electrodes (100 mm × 50 mm × 2 mm) were inserted vertically at fixed intervals of 3 cm and connected to a DC power supply (Figure 18).

Electrolysis was initiated, and 10 mL samples were collected at 0, 30, 60, and 90 min intervals. These samples were centrifuged at 5000 rpm for 3 min, and measurements were taken at the relevant wavelength using a UV-VIS spectrophotometer. The percentage removal (%R) of BB3 dye was calculated with Equation (1). During the experiments, the effects of initial dye concentration (2–20 mg/L), electrolysis time (15–90 min), shaking speed (100–180 rpm), medium pH (pH 4–10), and varying amounts (5, 10, 15 mL) of different electrolytes (NaCl, KCl) on the % removal values were also examined.

## 4. Conclusions

In this study, linden tree leaves (*Tilia* L.) were used as an adsorbent.

Their removal capacity for the BB3 cationic dye from aqueous media was investigated, and optimum operating conditions were examined. Additionally, BB3 dye was removed from the aqueous environment using the electrochemical method.

The performances of the two applied methods under ideal operating conditions were compared, thereby contributing to the comparative data on the two methods, which are currently limited in the literature.

The method using linden tree leaves (*Tilia* L.) as an adsorbent has proven effective in removing BB3 dyes from water. The optimal experimental conditions were determined to be: 50 mL of a 5 mg/L initial dye solution, 0.9 g of adsorbent, 60 min of contact time, 180 rpm agitation speed, 30 °C, and a pH of 10. The highest BB3 removal percentage achieved was 99.21% using this adsorption method.

In experiments using the electrocoagulation method, the optimal operating conditions were determined to be as follows: 1 L of a 15 mg/L dye solution, 15 mL of 0.2 M KCl added, 90 min of electrolysis time, 100 rpm agitation speed, and a pH of 10. Using the electrocoagulation method under these optimal conditions, the removal of BB3 was found to be 97.98%.

A comparison experiment in real water source applications using distilled water, Meriç River water, and tap water showed a high removal potential of 99.21% with distilled water. However, BB3% removal values have also been successfully achieved at a remarkable level in other water sources. Adsorption data were found to be compatible with the linear Langmuir model, yielding a correlation coefficient (r^2^) of 0.992. The highest adsorption capacity was calculated as 5.28 mg/g.

This study compared the effectiveness of adsorption and electrocoagulation methods in removing BB3 dye from water. The adsorption process utilized a natural, sustainable adsorbent made from linden leaves, achieving a 99.21% removal and potentially offering a cost-effective solution. Although electrocoagulation achieved a 97.98% removal, managing its accumulated sludge can be expensive. On the other hand, the adsorbent used in the adsorption method can be regenerated and reused, eliminating the need for disposal. Generally, adsorption is suitable for low-cost, eco-friendly treatment, while electrocoagulation provides rapid and highly efficient removal for large-volume processes.

Furthermore, it has been demonstrated that Tilia leaves are not only beneficial for health but also effective in removing toxic dyes. In ongoing studies, we plan to repeat the experiments using different electrodes (such as Fe and Ni) mentioned in the literature, instead of the Al electrode used in electrocoagulation. Future goals of this study include testing the effectiveness of both methods in removing other toxic dyes. This will help develop natural and efficient wastewater treatment techniques.

## Figures and Tables

**Table 1:** Langmuir and Freundlich isotherm constants for the adsorption of BB3 on linden leaves.

	Langmuir Isotherm			Freundlich Isotherm		
	*K_L_* (mg/L)	*q_max_* (mg/g)	*r* ^2^	*K_f_* (mg/L)	1/*n*	*r* ^2^
Linear Non-Linear	−5.837	5.283	0.992	1.544	5.242	0.993

**Table 2:** The removal data about the BB3 dye using different adsorbent materials.

Adsorbent	Adsorption Capacities (mg g^−1^)	Adsorption Model	References
	BB3		
Linden Tree Leaves (*Tilia* L.)	5.28	Langmuir	This Study
Silybum marianum (SLM) Stem-Natural	13.96	Langmuir	[23]
Silybum marianum (SLM) Stem-800 °C	36.80	Langmuir	[23]
Mulberry leaves (*Morus nigra* L.)	10.30	Langmuir	[24]
Raw cedar sawdust	47.62	Langmuir	[25]
Quarternized sugarcane bagasse	37.59	Freundlich	[26]
Sulfuric acid-activated montmorillonit	303–64.53	Langmuir–Freundlich	[27]
PANI	47.97	Langmuir	[28]
Chitosan-based adsorbent	166.5	Langmuir	[29]

**Table 3:** Studies using different electrodes are found in the literature.

Wastewater	Removal Efficiency (%)	Electrode Material	References
Textile Industry (BB3)	97.98	Al	This Study
Recycled fiber	94.91	Fe	[30]
Recycled fiber based	75.03	Al	[31]
Paper mill	83.00	Al	[32]
Petroleum refinery	52.00	Stainless Steel	[33]
Paper mill	>95.00	Al/Fe	[34]

## Data Availability

The original contributions presented in this study are included in the article. Further inquiries can be directed to the corresponding author.
